# Changes in Cardiopulmonary Reserve and Peripheral Arterial Function Concomitantly with Subclinical Inflammation and Oxidative Stress in Patients with Heart Failure with Preserved Ejection Fraction

**DOI:** 10.1155/2014/917271

**Published:** 2014-02-27

**Authors:** Damien Vitiello, François Harel, Rhian M. Touyz, Martin G. Sirois, Joel Lavoie, Jonathan Myers, Anique Ducharme, Normand Racine, Eileen O'Meara, Mathieu Gayda, Malorie Chabot-Blanchet, Jean Lucien Rouleau, Simon de Denus, Michel White

**Affiliations:** ^1^Research Center, Montreal Heart Institute, Université de Montréal, 5000 Belanger Street East, Montreal, QC, Canada H1T 1C8; ^2^Departments of Medicine and Pharmacology, Faculty of Medicine, Université de Montréal, Montreal, QC, Canada; ^3^Institute of Cardiovascular and Medical Sciences, University of Glasgow, Glasgow, UK; ^4^The Kidney Research Center, Ottawa Hospital Research Institute, University of Ottawa, Ottawa, ON, Canada; ^5^Palo Alto VA Health Care System, Stanford University, Palo Alto, CA, USA; ^6^Cardiovascular Prevention and Rehabilitation Centre, Montreal Heart Institute, Montreal, QC, Canada; ^7^Coordinating Center, Montreal Heart Institute, Montreal, QC, Canada; ^8^Faculty of Pharmacy, Université de Montréal, Montreal, QC, Canada

## Abstract

*Background*. Changes in cardiopulmonary reserve and biomarkers related to wall stress, inflammation, and oxidative stress concomitantly with the evaluation of peripheral arterial blood flow have not been investigated in patients with heart failure with preserved ejection fraction (HFpEF) compared with healthy subjects (CTL). *Methods and Results*. Eighteen HFpEF patients and 14 CTL were recruited. Plasma levels of inflammatory and oxidative stress biomarkers were measured at rest. Brain natriuretic peptide (BNP) was measured at rest and peak exercise. Cardiopulmonary reserve was assessed using an exercise protocol with gas exchange analyses. Peripheral arterial blood flow was determined by strain gauge plethysmography. Peak VO_2_ (12.0 ± 0.4 versus 19.1 ± 1.1 mL/min/kg, *P* < 0.001) and oxygen uptake efficiency slope (1.55 ± 0.12 versus 2.06 ± 0.14, *P* < 0.05) were significantly decreased in HFpEF patients compared with CTL. BNP at rest and following stress, C-reactive-protein, interleukin-6, and TBARS were significantly elevated in HFpEF. Both basal and posthyperemic arterial blood flow were not significantly different between the HFpEF patients and CTL. *Conclusions*. HFpEF exhibits a severe reduction in cardiopulmonary reserve and oxygen uptake efficiency concomitantly with an elevation in a broad spectrum of biomarkers confirming an inflammatory and prooxidative status in patients with HFpEF.

## 1. Introduction

Heart failure with preserved ejection fraction (HFpEF) is associated with a decrease in cardiopulmonary reserve leading to significant maladaptive changes in peripheral arterial [1] and muscular functions [2]. Cardiopulmonary reserve and oxygen uptake efficiency are both decreased in chronic heart failure with reduced ejection fraction (HF-rEF) [3, 4]. Other small studies have demonstrated that the oxygen uptake efficiency slope (OUES) is decreased in chronic HF patients [5] and in older patients with HFpEF [6].

HFpEF is characterized by an increase in some biomarkers related to neurohumoral activation [7, 8]. Previous investigations have reported significant differences between patients with HFpEF versus HF patients with reduced ejection fraction [7, 8] such as lower N-terminal prohormone of brain natriuretic peptide (NT-proBNP) in HFpEF. The characterization of changes in biomarkers at rest and following peak exercise has not been fully addressed in this form of HF. Similarly, disorders of endothelial function and peripheral arterial blood flow have been a matter of controversies in patients with HFpEF [1, 9–12]. No investigations have studied the changes in biomarkers related to LV wall stress, subclinical inflammation, and oxidative stress concomitantly with the evaluation of cardiopulmonary reserve and peripheral arterial function in HFpEF compared with healthy subjects.

The primary objective of this study was to investigate the changes cardiopulmonary reserve and peripheral arterial function, and biomarkers related to neurohumoral activation, inflammation, and oxidative stress in patients with HFpEF compared with healthy subjects. The secondary objective was to explore the relationship between biomarkers and functional capacity.

## 2. Methods

### 2.1. Study Population

This study was a prospective nonrandomized investigation including both patients with HFpEF and healthy subjects. Eighteen (18) patients and 14 healthy subjects were recruited. Patients were included in the HFpEF group if they had New York Heart Association (NYHA) classes II and III symptoms and if they had a left ventricle ejection fraction (LVEF) ≥50% measured by echocardiography within the 12 months prior to enrolment in the study. The diagnosis of HFpEF was confirmed by the presence of at least one abnormality on the screening echocardiography consistent with this condition such as atrial dilatation, left ventricle (LV) concentric remodeling or hypertrophy, and/or evidence of diastolic dysfunction by Doppler studies. LV volumes and filling rates were further assessed by radionuclide ventriculography at the beginning of the study. Patients with symptomatic hypotension (systolic blood pressure (SBP) < 90 mmHg) or poorly controlled hypertension (SBP ≥ 160 and/or diastolic blood pressure > 90 mmHg) were excluded. Similarly, patients with severe chronic pulmonary disease limiting exercise capacity, severe renal failure (creatinine > 250 *μ*mol/L), or significant liver dysfunction (transaminases ≥ 3-fold upper normal values) were excluded. Healthy subjects were included if they presented with no significant medical conditions and were on no medication at the time of assessment. Subjects or patients presenting with acute or active chronic inflammatory conditions were excluded from this study. All patients and healthy subjects provided written informed consent before undergoing any study-related procedures. The investigation conforms to the principles outlined in the Declaration of Helsinki. The study was approved by the Montreal Heart Institute—Research Scientific and Ethics Committees.

### 2.2. Maximal Exercise Testing

The maximal exercise test was performed on a treadmill using a RAMP protocol [13]. Gas exchange parameters were measured breath by breath during testing, and then averaged every 15 seconds for minute ventilation (VE, L/min), O_2_ uptake (VO_2_, L/min), and CO_2_ production (VCO_2_, L/min) using an automated gas analyzer system (Oxycon Pro, Hoechberg, Germany) [14]. Heart rate and manual brachial blood pressure were recorded before the test and at 2-minute intervals during exercise and recovery. Criteria for maximal effort were the attainment of the primary maximal criteria, a leveling off of oxygen uptake (<150 mL/min) despite increased intensity or one of the three secondary maximal criteria: (1) a respiratory exchange ratio >1.05, (2) inability to maintain walking, and (3) patient exhaustion due to fatigue or other clinical symptoms (dyspnea, ECG, and/or blood pressure abnormalities) [14]. The average value of the VO_2_ recorded during the last 15 seconds of exercise was considered as the peak oxygen uptake (VO_2_ peak), and VE/VCO_2_ slope was also determined. The oxygen uptake efficiency slope (OUES) was calculated during exercise using the slope of the relation VO_2_ and the log of ventilation as previously reported [15]. The heart rate recovery (HRR) was measured at 1 (HRR 1) and 2 (HRR 2) minutes following the termination of exercise.

### 2.3. Biomarkers Measurements

Venous blood samples were taken after semisupine rest for at least 15 minutes from both experimental populations under fasting state in the morning. Serum samples were centrifuged (1500 g, 15 min, 4°C) and immediately frozen at −80°C. Blood tests were performed in the resting state for all parameters and within 2 minutes following peak exercise for the brain natriuretic peptide (BNP).

Neurohumoral activation was assessed by plasma levels of both BNP and NT-proBNP. These two biomarkers were measured by electrochemiluminescence immunoassay using the Roche BNP and proBNP assays (Roche Diagnostics, Mannheim, Germany) on the Elecsys 2010 analyzer (Roche Diagnostics). Serum high-sensitivity C-reactive protein (hsCRP) was measured using the Dade Behring CardioPhase hsCRP assay (Siemens Healthcare Diagnostics Products, Marburg, Germany) on the BN ProSpec Nephelometer (Siemens Healthcare Diagnostics Products). Plasma level of thiobarbituric acid reactive substances (TBARS) was measured colorimetrically as previously described [16]. Plasma levels of interleukin-6 (IL-6) and 8-epi-prostaglandin F2*α* were analyzed by ELISA using the R&D Systems kits (Minneapolis, MN, USA).

### 2.4. Strain Gauge Plethysmography (SGP)

All measurements of blood flow were performed 2 hours after morning medications. Forearm basal arterial flow was assessed using the strain gauge plethysmography (SGP) methods as previously described [17]. Briefly, all subjects sat with their arms resting in a supine position on supports positioned above the level of the heart. Venous cuffs were then connected to automatic pneumatic inflators (Hokanson, E-20 rapid cuff inflator; Bellevue, WA) set to 50 mmHg and calibrated strain gauges were placed around both forearms and connected to a plethysmograph (Hokanson, model EC-4, Bellevue, WA). Baseline flow measurements were performed before and after a 240-second period of arterial occlusion. Arterial inflow was calculated by determining the upslope of strain gauge signals calculated using a linear regression model.

### 2.5. Statistical Analyses

Continuous baseline characteristics are expressed as mean ± standard deviation and categorical variables as frequencies and percentages. A logarithmic transformation was applied to variables showing a lognormal distribution. The proportion of male was compared between groups with a Chi-square test and continuous baseline characteristics were compared using a Student's *t*-test. All measurements including parameters of cardiopulmonary function, biomarkers, and arterial blood flow were analyzed using ANCOVA or repeated measures ANCOVA including age as a covariate to control for its potentially confounding effect. Contrasts between groups were performed at each time point in the repeated measures model. Basal and hyperemic arterial blood flows were summarized by computing area under the curve. Results are expressed as adjusted means ± standard errors or adjusted geometric means. To evaluate whether biomarkers influenced aerobic capacity, Pearson's correlations were performed. A *P* value < 0.05 was considered statistically significant. Statistical analyses were performed using the SAS software (version 9.2 or higher).

## 3. Results

A total of 32 subjects were recruited for this study including 18 patients with HFpEF and 14 healthy subjects. The clinical characteristics of the study population are shown in Table 1. The majority of patients exhibited systemic hypertension as a cause of HF. Of the patients studied, 83% were in NYHA class II symptoms at the time of admission. All HFpEF patients exhibited a larger LV end-diastolic volume and a shorter peak filling rate (PFR) with a higher time to PFR compared with the healthy subjects confirming a significant diastolic dysfunction in our patients. LVEF was higher in patients with HFpEF. The majority of patients (67%) were treated with an angiotensin II receptor blocker (ARBs) and 50% received a beta-blocker.

Exercise and gas exchange parameters are presented in Table 2. All patients and healthy subjects performed a maximal effort as evidenced by a respiratory exchange ratio >1.05 (data not shown). Exercise duration and peak METS achieved were significantly lower in patients with HFpEF compared with healthy subjects. The OUES was reduced by 31% in our patients. Similarly, peak VO_2_ and the VE/VCO_2_ slope were significantly decreased by 41% and increased by 15%, respectively. HRR at 1 and 2 min after the termination of exercise were significantly lower in patients compared with the healthy subjects.

**Figure 1:**
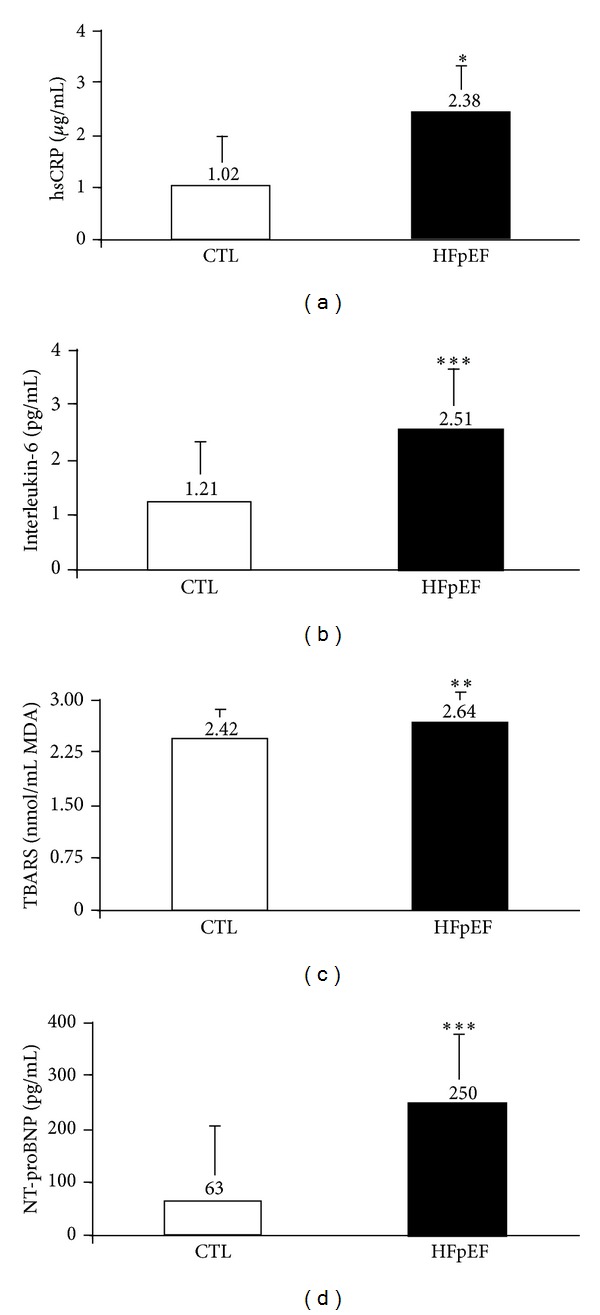
Circulating biomarker levels for patients with HFpEF versus healthy control subjects. NT-proBNP: N-terminal prohormone of brain natriuretic peptide; hsCRP: high-sensitivity C-reactive protein; TBARS: thiobarbituric acid reactive substances. Values are expressed as adjusted geometric mean or adjusted mean ± error. Significantly different from HFpEF values: *P < 0.05; **P < 0.01; ***P < 0.001.

**Figure 2:**
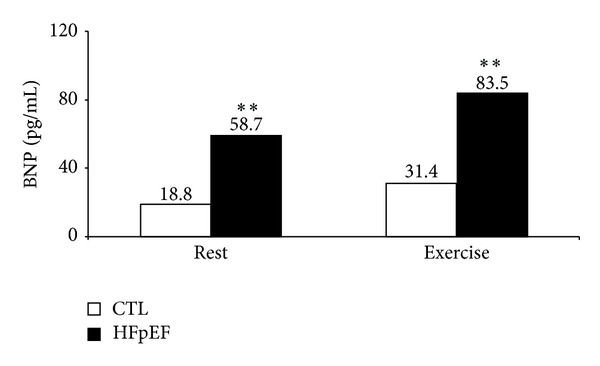
Changes in brain natriuretic peptide at rest and at peak exercise in patients with HFpEF versus healthy subjects. BNP: brain natriuretic peptide. Values are expressed as adjusted geometric mean. Significantly different from HFpEF values: **P < 0.01.

Biomarkers data for the study population are presented in Figures 1 and 2. Plasma levels of hsCRP (*P* < 0.05), TBARS (*P* < 0.01), and 8-epi-prostaglandin F2*α* (*P* < 0.05) were significantly increased in patients with HFpEF compared with healthy subjects. The patients exhibited a 4-fold increase in NT-proBNP (*P* < 0.001) (Figure 1) and a 3-fold increase in BNP plasma concentrations (*P* < 0.01) in resting state (Figure 1). This difference persisted at peak exercise (Figure 2).

**Figure 3:**
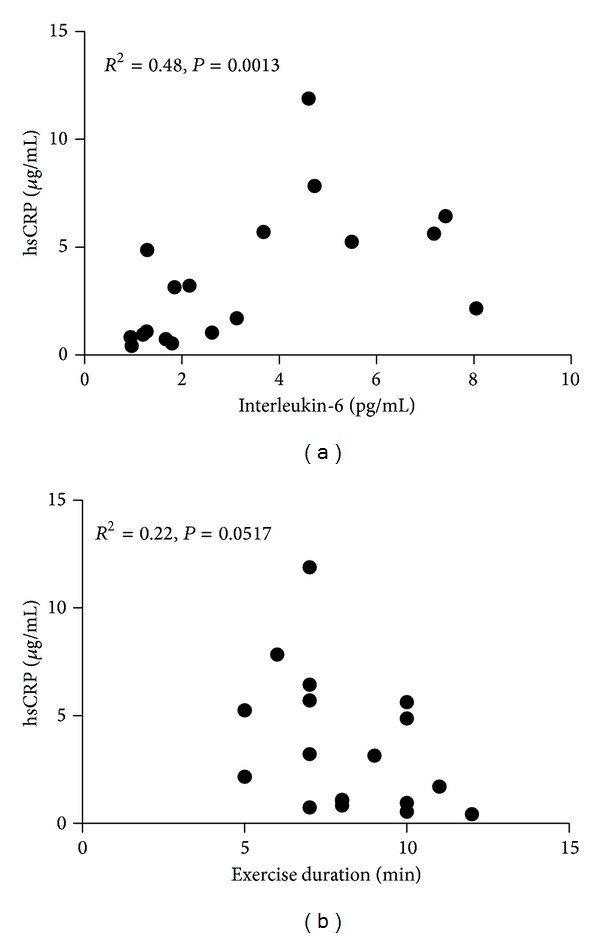
Relationships between selected inflammatory biomarkers and exercise duration in patients with HFpEF. hsCRP: high-sensitivity C-reactive protein; *R*²: coefficient of determination.

The relationships between biomarkers with selected exercise and biochemistry parameters are presented in Table 3 and Figure 3. Significant relationships were observed between BNP, hsCRP, IL-6, and 8-epi-prostaglandin F2*α* and peak VO_2_ and HRR 2 (Table 3). There was also a modest but significant relationship between hsCRP and IL-6 and between hsCRP and exercise duration in the HFpEF population (Figure 3).

**Figure 4:**
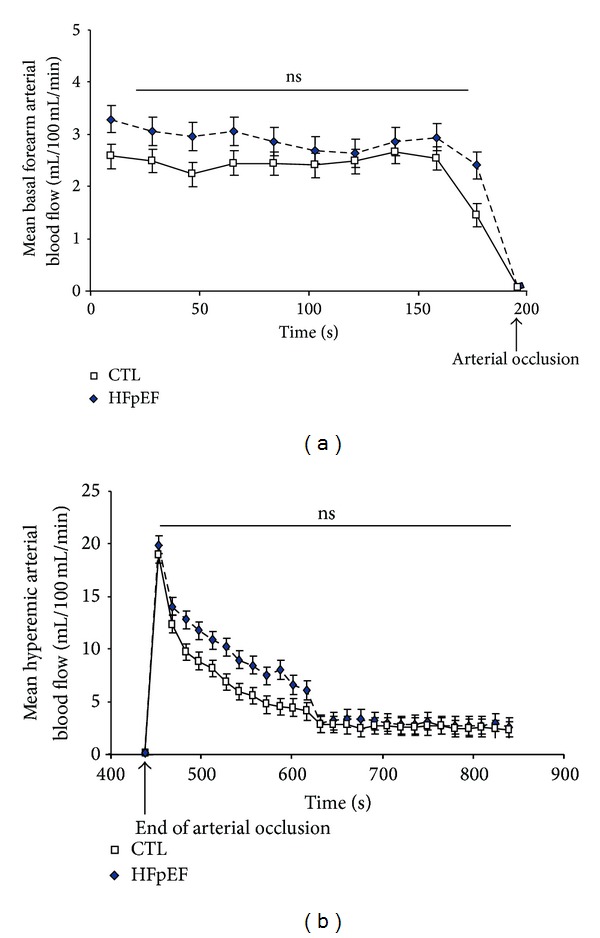
Changes in basal (a) and hyperemic (b) arterial blood flow for patients with HFpEF versus healthy control subjects. Values are expressed as adjusted mean ± standard error.

Peripheral arterial flows in resting state and following arterial occlusion are presented in Figure 4. Basal peripheral arterial forearm blood flow was not statistically different in the study population as demonstrated by the area under the curve (AUC) in HFpEF patients compared with healthy subjects (resp., 523 ± 70 versus 386 ± 41, NS) (Figure 4(a)). No difference in the hyperemic response was observed between the two groups (Figure 4(b)).

## 4. Discussion

In this study we reported a significant reduction in aerobic capacity and oxygen uptake efficiency in ambulatory patients with HFpEF. We also reported a significant increase in some biomarkers related to subclinical inflammation and oxidative stress. Both BNP and NT-proBNP were significantly elevated at rest with a similar magnitude of BNP increase at peak exercise in both patients and healthy subjects. In addition, we observed some significant relationship between peak aerobic capacity and HRR following exercise with BNP, IL-6, and 8-epi-prostaglandin F2*α*. We observed no significant differences in basal and posthyperemic blood flow in HFpEF patients compared with healthy subjects.

Previous investigations have reported a significant reduction in functional and peak aerobic capacities in patients with HFpEF [2, 18–20]. Here we reported a decrease in peak VO_2_ of 37% in patients with HFpEF compared with controls. This magnitude of decrease is in agreement with the overall decrease of 40% reported by other investigators [2, 18–20]. In addition, we observed a 30% reduction in the OUES in HFpEF patients compared with healthy control subjects. These changes are consistent with previous reports [2, 20] showing significant decrease in cardiopulmonary reserve and abnormal ventilator function in these patients.

Previous investigations have shown an increase in selected biomarkers such as IL-6 and NT-proBNP in patients with HFpEF [7, 8, 21]. Our findings confirm our former observations and data from other investigators showing significant increases of the C-reactive protein and IL-6 and demonstrating a significant proinflammatory state in these patients [7, 21, 22]. In addition to earlier studies [23, 24], we reported a 3-fold increase in BNP at rest which was maintained at peak exercise in HFpEF patients. The similar magnitude of BNP increase at peak exercise for both HF and health subjects patients suggests a preservation of wall stress during exercise in patients with HFpEF. Here we also reported a significant increase in biomarkers related to oxidative stress in patients with HFpEF compared with healthy subjects. These findings have not been reported before. Indeed, two biomarkers of oxidative stress including TBARS and 8-epi-prostaglandin F2*α* were both significantly increased, confirming a prooxidative state in these patients. Previous investigations have reported a role of oxidative stress in the pathophysiology of HF [25, 26]. Other observations have reported a detrimental effect of oxidative stress on the degradation of cardiac extracellular matrix degradation in humans [27] and on the cardiac contractility in mice [28]. The role of biomarker changes and specially those related to subclinical inflammation and oxidative stress on the pathophysiology of HFpEF remain unknown. We further explored the relationships between selected clinical and functional parameters with some biomarkers in our study population. We reported a significant relationship between peak VO_2_ and HRR at 2 minutes with BNP, 8-epi-prostaglandin F2*α*, hsCRP, and IL-6 in the overall population. This suggests a significant relationship between inflammation and autonomic regulation with functional capacity in HFpEF patients. These observations are in agreement with previous studies showing a relationship between sympathetic and parasympathetic tones and regulation of inflammation in chronic HF patients [29] and in a canine pacing model of HF [30]. Additional investigations are needed to confirm these findings.

Here, we reported no significant differences in basal and posthyperemic peripheral arterial blood flow in patients with HFpEF compared with healthy subjects. Abnormal endothelial function is associated with a decreased aerobic capacity in high risk patients [9] and in patients with HF with decreased LVEF [12]. There has been little data regarding the changes in peripheral arterial blood flow at rest and following stress in patients with HFpEF. A previous investigation reported a decrease in leg blood flow at rest and following exercise [1]. In contrast, other clinical studies reported no difference in leg flow-mediated dilation [11] or in brachial artery flow-mediated dilation [10] following submaximal exercise compared with healthy subjects. In that same study, no significant relationship between the reduction in peak VO_2_ and brachial artery flow-mediated dilation has been reported beyond the effect of aging [10]. The differences between a previous study [1] and our data may be explained by some clinical differences in the patient population and methodological approaches. First, the etiology of HF was different with some patients presenting dyspnea because of bronchial asthma in the latter study [1]. Most importantly, the rate of use of angiotensin-II modulating agents was 73% in the current study as opposed to 40% on average in previous publications [1, 10]. The high proportion of use of ARBs (i.e., 67%) may have contributed to attenuate the changes in basal and posthyperemic blood flow in our patients [31, 32]. Finally, we used SGP as opposed to magnetic resonance [1, 11] or brachial artery flow-mediated dilation [10] methods. Contrary to these techniques, we mechanically assessed the increase in forearm volume after the cuff deflation using calibrated strain gauges connected to a plethysmograph. This technique correlates well with the near-infrared spectroscopy for noninvasive assessment of arterial forearm flow [17]. Nevertheless SGP may not be sensitive enough to detect small changes in microvascular function in HFpEF patients.

Several factors may limit the conclusions of this study. Firstly, the population of patients was older than the control population. However, to minimize the impact of age on our observations ANCOVA analyses were computed using age as a covariate. Also no investigations have reported any effect of age on biomarkers and functional parameters in patients with symptomatic HF caused by preserved ejection fraction. Secondly, the sample size was small. Despite this, our study population was fairly homogenous allowing small variance and significance in most of the parameters studied. Thirdly chronic use of ARBs may have significantly impacted our findings on forearm blood flow data. Finally, we only measured plasma level of BNP at peak exercise. The inclusion of other biomarkers may have provided additional insights on the mechanisms involved with exercise limitations in these patients.

In conclusion, this study demonstrates that ambulatory patients with HFpEF exhibit a significant reduction in cardiopulmonary reserve and oxygen uptake efficiency concomitantly with an elevation in broad spectrum of biomarkers confirming a proinflammatory and a prooxidative status in these patients. The relationship between some biomarkers of inflammation and oxidative stress suggest a role of these processes on functional capacity in these patients. The role of biomarkers and the assessment of peripheral arterial function by multimodality techniques deserve further investigations.

## Figures and Tables

**Table 1:** Baseline characteristics of the study population.

Clinical variables	HFpEF patients (*n* = 18)	Healthy controls (*n* = 14)
Age (years)	70.7 ± 8.9*	61.7 ± 9.9
Male	5 (28%)	6 (43%)
Heart rate (bpm)	60.8 ± 8.9*	70.2 ± 7.7
Systolic blood pressure (mmHg)	125 ± 16	126 ± 18
Diastolic blood pressure (mmHg)	72.4 ± 8.2	76.3 ± 7.1
Duration of heart failure (months)	22.3 ± 24.2	—
NYHA functional class		
II	15 (83%)	0 (0%)
III	3 (17%)	0 (0%)
Etiology of heart failure		
Ischemic	3 (17%)	0 (0%)
Hypertension	15 (83%)	0 (0%)
Laboratory values		
Haemoglobin (mg/L)	131 ± 13**	145 ± 12
Serum creatinine (*μ*mol/L)	106 ± 43*	79.7 ± 15.4
Medications		
ACE inhibitors	1 (6%)	0 (0%)
ARBs	12 (67%)	0 (0%)
Beta-blockers	9 (50%)	0 (0%)
Radionuclide angiography		
LVEF (%)	57.5 ± 7.0*	52.1 ± 6.2
LVEDV (mL)	118.3 ± 33.3*	98.0 ± 19.1
PFR (EDV/s)	1.95 ± 0.50*	2.34 ± 0.42
TPFR (ms)	182 ± 53*	147 ± 40

ACE: angiotensin-converting enzyme; ARBs: angiotensin II receptor blockers; LVEDV: left ventricle end-diastolic volume; PFR: peak filling rate of the left ventricle; TPFR: time to peak filling rate of the left ventricle; LVEF: left ventricular ejection fraction; NYHA: New York Heart Association. Continuous variables are expressed as mean ± standard deviation and categorical variables as frequencies and percentages. *P < 0.05; **P < 0.01.

**Table 2:** Exercise haemodynamics and gas exchange parameters for the study population.

Stress variables	HFpEF patients (*n* = 18)	Healthy controls (*n* = 14)
Duration (min)	8.33 ± 0.48*	10.36 ± 0.55
Maximal energy expenditure (METS)	4.81 ± 0.21***	8.07 ± 0.48
Peak exercise heart rate (bpm)	106 ± 5***	162 ± 6
Peak exercise systolic blood pressure (mmHg)	158 ± 6*	180 ± 7
Peak exercise diastolic blood pressure (mmHg)	74.1 ± 1.9	79.6 ± 2.2
Peak VO_2_ (mL/kg/min)	12.0 ± 0.44***	19.1 ± 1.07
% of VO_2_ predicted for age	87 ± 5***	123 ± 6
Heart rate recovery at 1 min (bpm)	17.0 ± 2.2*	24.4 ± 2.6
Heart rate recovery at 2 min (bpm)	32.1 ± 3.1***	50.0 ± 3.6
VE/VCO_2_ slope	33.6*	29.3
OUES	1.55 ± 0.12*	2.06 ± 0.14

METS: metabolic equivalent tasks; OUES: oxygen uptake efficiency slope; VCO_2_: exhale carbon dioxide; VE: ventilation; VO_2_: oxygen uptake. Values are expressed as adjusted mean ± standard error or adjusted geometric mean. *P < 0.05; ***P < 0.001. For the VE/VCO_2_ slope variable, there was a significant interaction age ∗ group. In this table, we present the adjusted geometric means for an age of 68 years (median value) which is the closest age compared with our HFpEF patients. For *Q*1 (61 year old), there was no significant difference between HFpEF patients and healthy control subjects (30.9 versus 29.7, *P* = 0.57). For *Q*3 (75 year old), there was a significant difference between HFpEF and healthy control subjects (36.7 versus 28.9, *P* < 0.01).

**Table 3:** Correlations between biomarkers and peak VO_2_ for the study population.

	Peak VO_2_	HRR 2	BNP	hsCRP	IL-6	8-epi-PG-F2*α*	TBARS
Pearson correlation coefficients (*P* values)							
Peak VO_2_	1						
HRR 2	0.71***	1					
BNP	−0.66***	−0.57***	1				
hsCRP	−0.40*	−0.49**	0.45*	1			
IL-6	−0.63***	−0.57***	0.70***	0.67***	1		
8-epi-PG-F_2α_	−0.41*	−0.38*	0.44*	0.21	0.55**	1	
TBARS	−0.22	−0.19	0.26	0.39*	0.41*	0.43*	1

BNP: brain natriuretic peptide; HRR2: heart rate recovery at 2 min following the end of exercise; hsCRP: high-sensitivity C-reactive protein; IL-6: interleukin-6; TBARS: thiobarbituric acid reactive substances; VO_2_: oxygen consumption; 8-epi-PG-F2*α*: 8-epi-prostaglandin F2*α*. *P < 0.05; **P < 0.01; ***P < 0.001.
